# Suppression Subtractive Hybridization Reveals Transcript Profiling of Chlorella under Heterotrophy to Photoautotrophy Transition

**DOI:** 10.1371/journal.pone.0050414

**Published:** 2012-11-29

**Authors:** Jianhua Fan, Yanbin Cui, Jianke Huang, Weiliang Wang, Weibo Yin, Zanmin Hu, Yuanguang Li

**Affiliations:** 1 State Key Laboratory of Bioreactor Engineering, East China University of Science and Technology, Shanghai, People’s Republic of China; 2 Institute of Genetics and Developmental Biology, Chinese Academy of Sciences, Beijing, People’s Republic of China; University of Connecticut, United States of America

## Abstract

**Background:**

Microalgae have been extensively investigated and exploited because of their competitive nutritive bioproducts and biofuel production ability. *Chlorella* are green algae that can grow well heterotrophically and photoautotrophically. Previous studies proved that shifting from heterotrophy to photoautotrophy in light-induced environments causes photooxidative damage as well as distinct physiologic features that lead to dynamic changes in *Chlorella* intracellular components, which have great potential in algal health food and biofuel production. However, the molecular mechanisms underlying the trophic transition remain unclear.

**Methodology/Principal Findings:**

In this study, suppression subtractive hybridization strategy was employed to screen and characterize genes that are differentially expressed in response to the light-induced shift from heterotrophy to photoautotrophy. Expressed sequence tags (ESTs) were obtained from 770 and 803 randomly selected clones among the forward and reverse libraries, respectively. Sequence analysis identified 544 unique genes in the two libraries. The functional annotation of the assembled unigenes demonstrated that 164 (63.1%) from the forward library and 62 (21.8%) from the reverse showed significant similarities with the sequences in the NCBI non-redundant database. The time-course expression patterns of 38 selected differentially expressed genes further confirmed their responsiveness to a diverse trophic status. The majority of the genes enriched in the subtracted libraries were associated with energy metabolism, amino acid metabolism, protein synthesis, carbohydrate metabolism, and stress defense.

**Conclusions/Significance:**

The data presented here offer the first insights into the molecular foundation underlying the diverse microalgal trophic niche. In addition, the results can be used as a reference for unraveling candidate genes associated with the transition of *Chlorella* from heterotrophy to photoautotrophy, which holds great potential for further improving its lipid and nutrient production.

## Introduction

Green algae from the genus *Chlorella* are single-celled, microscopic plants that grow in fresh water and are believed to have existed for billions of years [1]. *Chlorella* is considered one of the most powerful “superfoods” known to man and is renowned for their incredible nutrient content and qualities [2]. *Chlorella* provides about 60% protein and it also contains an impressive range of vitamins and minerals, pigments, fatty acids, and growth factor. It is also become particularly promising in the development of second-generation biofuels because of its ability to accumulate high lipid content either under heterotrophic and phototrophic cultivation [3], [4].

The conventional commercial production of *Chlorella* biomass is typically accomplished in open ponds via photoautotrophy. Although a high demand exists in the market, its large-scale applications have been limited by the low productivity and high production cost of this purely phototrophic cultivation model. Thus, we investigated a novel cultivation strategy called “sequential heterotrophy-dilution-photoinduction” (SHDP) to develop a cost-effective, scaled-up culture method for *Chlorella* biomass and algal oil production [5]. Three *Chlorella* species, *C. pyrenoidosa*, *C. vulgaris*, and *C. ellipsoidea*, were investigated to confirm the feasibility of this approach in indoor and outdoor experiments on the microalgal mass culture process. The *Chlorella* species were first cultivated heterotrophically to achieve high cell densities. After the glucose was consumed, the broth was diluted and transferred into a lighted environment for photoinduction. Using this strategy, the quality of the *Chlorella* biomass approximates that of cells cultivated photoautotrophically within several hours. Analysis of the cellular components and electron micrograph studies suggest that the shift from heterotrophic to light-induced growth causes a rapid decrease in cell carbohydrate and corresponding increases in chloroplast proteins, pigments, and lipids [5]. These advantages make the SHDP approach a potential method for the mass cultivation of heterotrophic microalgae for both algal health food and biofuel production. Similar results were also achieved using other algal species via two-step culture systems (transition from heterotrophy to photoautotrophy) with improvements in valuable cellular components [6]–[8]. However, little physiologic and biochemical information is available, especially regarding the major molecular events that occur in microalgal cells under diverse trophic transitions. Thus, the actual cause of intracellular components changes and the mechanism for the SHDP process still needs to be fully understood.

Photosynthetic organisms have evolved direct and indirect mechanisms for sensing and responding to environmental changes (e.g., salt, drought, temperature, and light stresses) [9]–[11]. Emerging genomic sequencing and systems biological approaches such as transcriptomics, proteomics, and metabolomics have become essential for understanding how organisms respond and adapt to changes in their environment [12], [13]. Regarding microalgae, numerous studies on the effects of stress conditions (e.g., nitrogen deprivation and sulfur deprivation) on microalgal lipid, hydrogen, or starch content have been previously carried out [14]–[19]. Nevertheless, the mechanisms of how microalgae evolved self-protective strategies against physiologic stress at the molecular level are mainly limited to model organisms such as *Chlamydomonas* and *Phaeodactylum*. High-throughput omics analyses for investigating microalgal growth and metabolism remain relatively unexplored, especially in species without genomic foundation, which leads to the lack of information on global genetic expression in response to different stresses. Therefore, unlike traditional studies that focus on general stress conditions in model microalgae, SHDP serves as a particular model for studying the molecular events that occur in heterotrophic algae to adapt and cope with unfavorable environments for survival and growth. In this study, we hypothesized that the cause of growth inhibition and cellular changes under heterotrophy to photoautotrophy transition is due to the sudden light irradiation induced photooxidative stress. We further hypothesized that heterotrophically metabolized *Chlorella* attempt to maintain a balance between energy supply and consumption to protect the cells from the detrimental effects of glucose starvation. Thus, it is important to first obtain information on differentially expressed genes which play an important role in the acquisition of nutrition transition tolerance of *Chlorella* under light stress and glucose starvation.

PCR-based suppression subtractive hybridization (PCR-SSH) is a powerful tool for identifying differentially expressed genes, including genes with relatively low abundance [20]. Express sequence tags (ESTs) generated from the high-throughput sequencing of randomly selected cDNA clones in SSH libraries have proven to be a sensitive, rapid, and cost-effective technique for obtaining information on genes that play an important role in environmental stress tolerance [21]. In the current work, both forward (light-induced cells versus heterotrophic cells) and reverse (heterotrophic cells versus light-induced cells) SSH libraries were constructed using the unsequenced microalga *C. pyrenoidosa*. It was cultured via the SHDP approach to identify upregulated and downregulated genes under sudden light stress and glucose starvation, by using the optimized culture medium and culture conditions in flask under heterotrophy or continues artificial light illumination (in consideration of the maximum biomass productivity with significant cellular components transformation, 1–3 g/l for photoinduction at a light intensity of 200–300 µmol/m^2^/s was the optimum condition). A total of 544 candidate genes were found to be involved in diverse biological processes, that facilitate an understanding of the molecular basis of microalgal stress tolerance and nutrition transition.

## Materials and Methods

### Algal Materials and Culture Conditions

*C. pyrenoidosa* was obtained from the Institute of Hydrobiology, Chinese Academy of Sciences (Wuhan, China), and purified aseptically. They were first grown heterotrophically in Endo medium at 28°C [22] and shaken at 150 rpm. Light-induced treatment was carried out using late log-phase heterotrophic cells once the glucose was depleted. Aliquots (50 ml) of algal cells were collected by centrifugation at 5,000 ×*g* for 5 min in the dark. After washing twice with sterilized water, the pellets were resuspended in fresh Sorokin–Krauss medium [23] at a final cell concentration of ∼2 g/l and then subjected to continuous light treatment under a light intensity of 250 µmol/m^2^/s. Both heterotrophic and light-induced cells from different time courses were harvested and preserved in liquid nitrogen for future use.

For comparison between different cultivation strategies, the heterotrophic grown cells were further investigated by inoculation into several 3 L air-lift flat panel photobioreactors (35 cm high, 24cm long and 4 cm wide) with a working volume of 2.5 L. The following four combinations were carried out for sampling: a) glucose starvation in the dark; b) photoautotrophic growth with 250 µmol/m^2^/s light illumination when glucose was exhausted; c) mixotrophic growth with 20 g/l glucose and 250 µmol/m^2^/s light treatment; d) heterotrophic growth with 20 g/l glucose in the dark. The lamps arranged in parallel on a vertical plane at both sides, were used as the external light source for a 48-h continuous irradiance. Aeration and mixing were achieved by sparging air with 1.0 vvm.

### Analytical Methods

The biomass concentration, protein and chlorophyll contents were measured using the methods described by Fan et al [5]. The maximum photochemical efficiency of photosystem II (Fv/Fm) and non-photochemical quenching (NPQ) of fresh alga cells were determined with Dual-PAM 100 (Walz, Germany) according to the operating procedures used by Gao et al [24]. All experiments were conducted in three independent biological replicates. The Fv/Fm and NPQ values were calculated as follows: *Fv/Fm = (Fm−Fo)/Fm*; *NPQ = (Fm−Fm’)/Fm’*.

Reactive oxygen species (H_2_O_2_ and hydroxyl radical) were detected by using biochemical methods following the instructions of corresponding reagent kits (Nanjing Jiancheng Bioengineering Institute, China). The activities of ROS scavenging enzymes, such as superoxide dismutase (SOD), catalase (CAT), and peroxidase (POD), as well as lipid peroxidation level in terms of malondialdehyde (MDA) contents were measured using the methods of Zhang and Kirkham [25]. The ROS content, activity of each enzyme and MDA content were all expressed on a soluble protein basis. Soluble protein concentration was determined using bicinchoninic acid (BCA) method (BCA protein assay kit, Sangon company, China).

### Total RNA and mRNA Isolation

Total RNA was extracted from the heterotrophic and light-induced *Chlorella* cells (2, 8, and 24 h from both heterotrophic and light-induced cells) using a Plant RNA Extraction Kit (Autolab, China). The quantity and quality of the isolated total RNA were assessed by electrophoresis on denatured agarose gel and by measuring the absorbance at 230, 260, and 280 nm. For SSH, equal amounts of total RNA from heterotrophic and light-induced *Chlorella* cells at each time point were mixed. The Poly(A)+ mRNA samples were purified from the mixed total RNA using PolyATract mRNA Isolation System (Promega, USA) according to the manufacturer’s instructions.

### Construction of the Subtracted cDNA Libraries

Double-stranded cDNA reverse transcribed from 3 µg of the mixed mRNA was used for suppression subtractive hybridization (SSH) using a PCR-Select cDNA Subtraction Kit (Clontech, California, USA). Both forward (cDNA obtained from light-induced cells as tester and cDNA obtained from heterotrophic cells as driver) and reverse (cDNA obtained from heterotrophic cells as tester and cDNA obtained from light-induced cells as driver) SSH libraries were constructed following the manufacturer’s instructions. The efficiency of the cDNA subtraction was evaluated by PCR using beta-actin primers (Table S1) performed on subtracted and unsubtracted cDNAs for 18,23, 28, 33, and 38 cycles. The subtracted cDNA was further cloned into the pGEM-T easy vector (Promega, Beijing, China) and was transformed into chemically competent TOPO 10 *Escherichia coli* cells (Tiangen, Beijing, China). The transformed cells were selected on Luria–Bertani agar plates containing ampicillin at 37°C for screening.

### Amplification of cDNA Inserts

PCR amplification of the cDNA inserts from colonies was conducted using the primers T7 (5′TAATACGACTCACTATAGGG3′) and SP6 (5′AGCTATTTAGGTGACACTATAG3′). The PCR products were electrophoresed on 1.2% agarose gel to confirm the presence and size of the inserts before screening and sequencing.

### DNA Sequencing and Data Analysis

More than 1,500 differentially expressed cDNA fragments randomly picked from each subtracted SSH library were sequenced on an ABI 3700 sequencer (Applied Biosystems, USA). The software SeqClean (http://compbio.dfci.harvard.edu/tgi/software/) was used to eliminate the vector and primer sequences, and for trimming of the low-quality segments. The CAP3 program [26] was used for contig assembly. The obtained nucleotide sequences were identified in the non-redundant (nr) protein database using the Blastx program [27] on the NCBI homepage (http://www.ncbi.nlm.nih.gov/BLAST). The ESTs with significant database threshold E-values of 10^−5^ or better were classified into putative functional categories according to their putative biological roles and molecular functions. All assembled contigs and singletons were further annotated using NCBI nucleotide (nt) databases, InterProScan [28], SwissProt (www.expasy.org/sprot), and other specialized databases like KEGG [29] and Clusters of orthologous groups (COGs, http://www.ncbi.nlm.nih.gov/COG).

### Semiquantitative and Real-time Quantitative RT-PCR Verification

Semiquantitative RT-PCR was performed to roughly and quickly validate the differential expression of each library. Total RNA was extracted from *Chlorella* cells collected at six different culture stages (2, 8, and 24 h for both heterotrophic and light-induced cells) according to the method described in Section 2.3. The RNA samples were treated with RNase-free DNase I (TaKaRa, Japan) to remove DNA contamination. The cDNA from each sample were synthesized using a ReverTra Ace -α- Kit (Toyobo, China) following the manufacturer’s protocol. The gene-specific primer pairs were designed from assembled unigenes using Primer Premier 5 (Table S1). The actin gene from *C. pyrenoidosa* was used as an internal control to normalize differences between the loading amounts of the template. All PCR reactions were carried out within a final volume of 20 µl containing 1 µl of cDNA template, 1.5 µl of 10 mM deoxyribonucleotide triphosphates, 2 µl of 10× PCR buffer (Mg^2+^ plus), 1 µl of each primer (10 µM), 0.2 µl of Taq polymerase (5 U/µl). The PCR parameters were set as follows: 28 cycles of 94°C for 30 s, appropriate annealing temperatures for 30 s, and 72°C for 1 min, with an additional initial 5 min denaturation at 94°C and a 5 min final extension at 72°C.

To further investigate the expression kinetics of the identified genes, real-time quantitative PCR was performed using SYBR Green Realtime PCR Master Mix (Toyobo, China) for 40 cycles, and the accumulation of fluorescent products was monitored using a C1000 Thermal cycler Real-Time PCR Detection System (Bio-Rad, CA, USA). All procedures were performed according to the manufacturers’ instructions. Table S2 summarizes the primer sequences used in this study. The 2^−ΔΔCT^ method was used to analyze the fold change in gene expression relative to control [30].

### Statistical Analysis

Data were expressed as mean ± SD. Statistical analysis was performed by one-way ANOVA followed by Newman–Keuls multiple comparison test (GraphPad Prism 5). *p-*values of less than 0.05 and 0.01 were taken to indicate a significant and extreme significant difference between groups respectively.

## Results

### Effect of Diverse Nutrition Transition on Growth and Physiological Features

**Figure 1:**
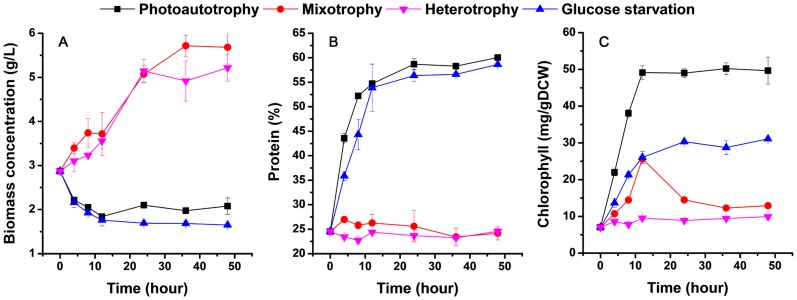
*Chlorella* growth and the cellular component temporal pattern characteristics under diverse nutrition transition. A: Biomass concentration; B: Protein content; C: Chlorophyll content. Error bars represent the mean ± standard deviation of three independent biological replicates.

**Figure 2:**
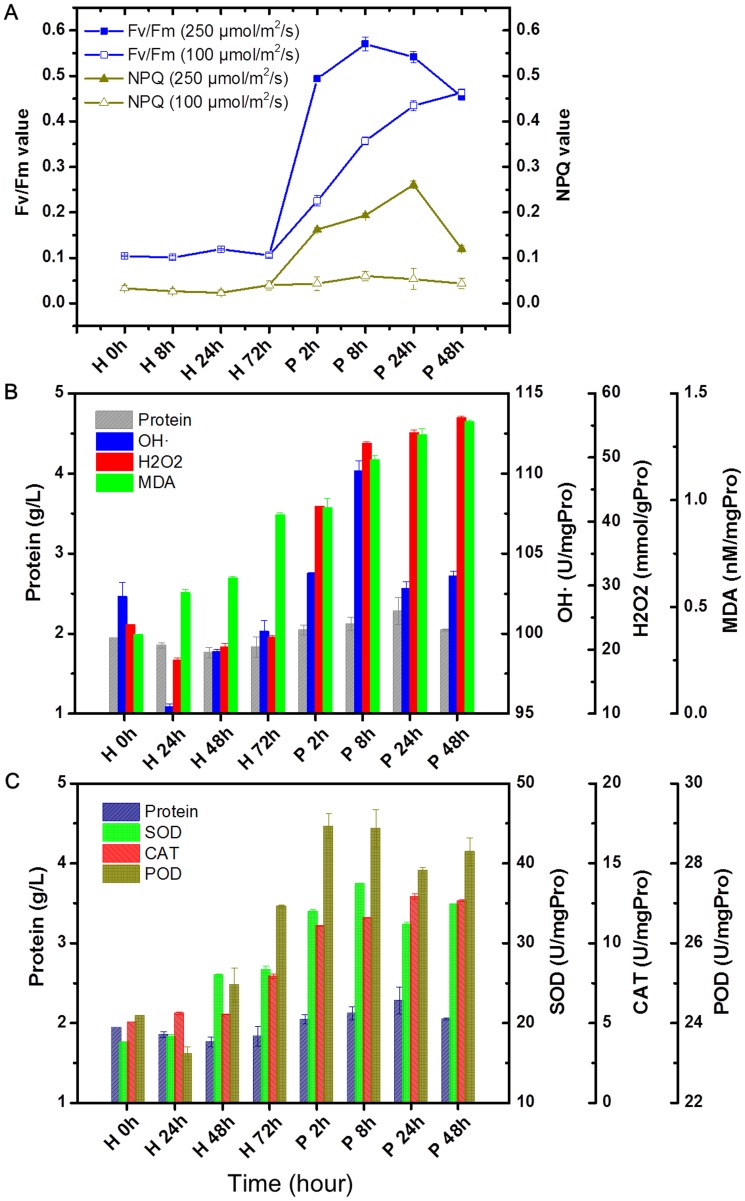
Time course changes on *Chlorella* physiological features under heterotrophy to photoautotrophy transition. A: Changes of photosynthesis fluorescence parameters on different light intensity; B: Production of ROS and lipid peroxidation; C: Activities of ROS scavenging enzymes. H: heterotrophy; P: photoautotrophy. Error bars represent the mean ± standard deviation of three independent biological replicates.

Different transition strategy of heterotrophy to photoautotrophy, mixotrophy, heterotrophy and glucose starvation were investigated to evaluate the effects on cell growth and physiological characteristics by batch mode operation, and the results are shown in Figure 1 and 2. The cultures for lack of glucose treatments (photoautotrophy and glucose starvation) exhibited a certain degree of weight loss (Figure 1A). Corresponding to this, photoautotrophy treatment resulted in the most drastic increment of cellular protein and chlorophyll contents, followed by glucose starvation (Figure 1B and 1C). With addition of glucose in the mediums (heterotrophy and mixotrophy), less cellular components variation but greatly increased biomass concentrations were observed, though slight increase of chlorophyll occurred in the mixotrophy treatment (Figure 1C). From the perspective of light, it could exacerbate the cellular change of dark-grown cells and shorten the induction period, making the quality of *Chlorella* cells reach the level of purely photoautotrophic cells rapidly (with 50% protein and 2% chlorophyll contents in general). Thus glucose and light may pull together effect on intracellular components and functional alterations, which maybe finally lead to differential gene expression response.

In order to determine how photosynthesis was affected by different culture modes transition, we also measured the maximum photochemical efficiency (Fv/Fm) and NPQ value to evaluate the photosynthesis efficiency and photoprotection capacity. The value of Fv/Fm is generally about 0.8 when algae cells are healthy [31], [32]. Figure 2A shows the time course change of Fv/Fm and NPQ in the heterotrophic process as well as medium (100 µmol/m^2^/s) and high (250 µmol/m^2^/s) light irradiation of photoautotrophy cultures. The heterotrophic-grown cells showed complete repressed photosynthesis as the photosynthetic apparatus was undeveloped in the dark. A maximum Fv/Fm of ca. 0.58 was measured at 8 h and then declined to ca. 0.45 two days after being transferred to high light environments, indicative of severe photoinhibition of photosynthesis. However, under medium light treatment, as the cultures proceeded, the cells gradually recovered from photoinhibition (Figure 2A). Simultaneously, the NPQ value of the high light treatment increased 2.79 to 4.92-fold compared to the medium light treatment in the first 48 h. Thus high light treatment led to decreased photosynthesis and increased photoprotection. In agree with probably increased photooxidative stress when transferred heterotrophic cells to photoautotrophic growth, the ROS concentration, ROS-induced lipid peroxidation (MDA content) and scavenging enzymes increased exponentially in the light-induced process, whereas only a slight increase in ROS and corresponding enzymes was detected in the heterotrophic cells under dark conditions (Figure 2B and 2C). The up-regulation of these features would potentially be caused by the increase of redox state, which perhaps explained intracellular metabolic changes follow photoinduction and glucose starvation.

### Characterization of the Subtracted cDNA Libraries

Two cDNA libraries were constructed by PCR-based SSH to obtain a large amount of ESTs for identifying differentially expressed genes involved in glucose starvation and light stress tolerance of *Chlorella* cells. Subtraction efficiency was validated using the abundances of the beta-actin gene transcripts (Figure S1). Differences in amplification patterns between the subtracted and unsubtracted cDNA samples indicative of successful subtraction were visually observed. The forward and reverse libraries consisted of approximately 3,000 positive clones. Before large-scale Sanger sequencing, 20 randomly selected clones were isolated and checked via colony PCR amplification to detect the presence of cDNA inserts and to estimate the size (Figure S2). A total of 1,573 clones (770 forward and 803 reverse) were isolated and sequenced, with the sizes of the inserts ranging from 120 bp to 1,000 bp (Figure S3). After removing the vector sequences and low-quality ESTs, the average available sequence was about 301 bp length (Figure S4). All the high-quality EST sequences have been deposited in the GenBank (accession numbers: dbEST JK744884–JK746369). The general characteristics of the two SSH libraries are provided in Table 1.

**Table 1:** Assembly and annotation statistics of the two SSH libraries.

Features	Forward library	Reverse library
**1. Sequencing and assembly quality**		
Total clones sequenced	770	803
High quality ESTs	722	767
Contigs	91	88
Singletons	169	196
Unigenes	260	284
Mean unigene length (nt)	328	285
**2. Unigene annotation**		
Supported by NCBI NT	165 (63.5%)	73 (25.7%)
Supported by NCBI NR	164 (63.1%)	62 (21.8%)
Supported by SwissProt	133 (51.2%)	41 (14.4%)
Supported by COGs	107 (41.2%)	23 (8.1%)
Supported by KEGG	141 (54.2%)	51 (18%)
Supported by InterProScan	161 (61.9%)	43 (15.1%)
Supported by Gene Ontology	149 (57.3%)	34 (12%)

### Contig Assembly, Functional Annotation, and Bioinformatics Analysis

The cleaned ESTs were assembled, which resulted in a total of 260 unigenes with 91 contigs/169 singletons from the light-induced cells and 284 unigenes with 88 contigs/196 singletons from the heterotrophically cultured cells (Table 1). Some unique genes were represented by multiple ESTs, as shown in Table S3 and S4, which included contigs with more than two nucleotide sequences. The properly assembled contigs and singletons were annotated using different tools (Blastn and Blastx) and databases (NCBI nucleotide and protein databases, InterProScan, KEGG, COGs, and SwissProt, see Tables S3 and S4 for details). Blastx annotation of the assembled unigenes demonstrated that 164 (63.1%) sequences from the forward library and 62 (21.8%) from the reverse library had significant similarities with the sequences in the NCBI nr database, whereas the rest of the unigenes had no homologues at the amino acid level (Table 1). The sequenced ESTs from the forward library were classified into 10 functional categories (Table 2). Energy metabolism (36, 5%); amino acid metabolism (80, 11%); DNA/RNA binding, transcription, and translation (347, 48%); protein folding, sorting, and degradation (16, 2%); and stress response (28, 4%) represented the largest portion of sequences. Similarly, the ESTs in the reverse library were divided into 11 groups (Table 3). The largest portions of the sequences were represented by energy metabolism (21, 3%); carbohydrate metabolism (12, 2%); DNA/RNA binding, transcription, and translation (12, 2%); protein folding, sorting, and degradation (16, 2%); and chlorophyll and polyketide biosynthesis (25, 3%). COGs–based functional prediction was also carried out to classify the differentially expressed genes (Figure S5).

**Table 2:** Functional classification of the differentially expressed genes in forward library.

Unigene ID	Length (bp)	No. ofESTs	Besthits	Identity	E-value	Annotation
**1. Energy Metabolism (36)**						
FYG062	507	2	AAB40979	54	5.00E-23	chlorophyll a–b binding protein of LHCI
FYG152	488	7	XP_001695467	80	1.00E-61	chlorophyll a–b binding protein of LHCII
FYG052	173	2	ABA01111	71	4.00E-11	chloroplast ATP synthase gamma subunit
FYG066	294	3	ABK24568	88	3.00E-38	fructose-1,6-bisphosphate aldolase
FYG177	196	1	CAA41635	69	6.00E-18	glutamate dehydrogenase (NADP+)
FYG056	486	4	ABA01139	83	2.00E-33	oxygen-evolving enhancer protein 1 of photosystem II
FYG242	635	1	XP_001694126	67	2.00E-42	oxygen-evolving enhancer protein 2 of photosystem II
FYG252	285	1	BAF97055	100	6.00E-19	Rubisco large subunit
FYG257	591	1	XP_001417684	59	2.00E-27	psaG, subunit V of photosystem I
FYG011	460	11	ACU32661	91	3.00E-11	Rubisco small subunit
FYG160	336	1	Q9SBN3	67	6.00E-26	plastocyanin oxidoreductase iron-sulfur protein
FYG248	169	1	ABD37953	86	5.00E-14	glyceraldehyde-3-phosphate dehydrogenase subunit A
FYG167	177	1	NP_192713	48	1.00E-07	short-chain dehydrogenase/reductase family protein
**2. Amino Acid Metabolism (80)**						
FYG129	521	80	XP_001697794	70	7.00E-61	diaminopimelate epimerase
**3. Nucleotide Metabolism (1)**						
FYG215	264	1	XP_001421179	66	2.00E-20	nucleoside diphosphate kinase
**4. Fatty Acid and Lipids Metabolism (1)**						
FYG250	320	1	ACF98530	88	1.00E-26	omega-6 fatty acid desaturase
**5. DNA/RNA Binding, Transcription and Translation (347, 342 ribosomal genes were not listed)**						
FYG179	336	1	XP_001700625	81	8.00E-15	transcription factor NAC-BTF3
FYG224	275	1	ABR26094	82	7.00E-18	retrotransposon protein
FYG193	389	1	XP_001702450	50	6.00E-16	RNA helicase
FYG042	910	2	AAV34148	93	1.00E-83	elongation factor 1-alpha
**6. Protein Folding, Sorting and Degradation (16)**						
FYG187	769	1	AAL15154	77	1.00E-102	serine protease
FYG069	291	15	NP_001054720	86	5.00E-34	ubiquitin family
**7. Transporter (3)**						
FYG150	279	1	NP_565182	42	2.00E-14	bile acid:sodium symporter protein
FYG048	195	2	XP_001493127	72	2.00E-12	chloride ion channels protein
**8. Cell Cycle and Motility (1)**						
FYG170	201	1	XP_001134549	74	7.00E-16	proliferation associated protein
**9. Response to Stress (28)**						
FYG201	257	1	XP_001693150	62	2.00E-11	glutathione peroxidase
FYG126	496	27	ABR01228	69	5.00E-30	thiazole biosynthetic enzyme
**10. Function Unknown (209)**						

Numbers in parentheses represent the number of ESTs categorized into each group.

**Table 3:** Functional classification of the differentially expressed genes in reverse library.

Unigene ID	Length (bp)	No. of ESTs	Best hits	Identity	E-value	Annotation
**1. Energy Metabolism (21)**						
RYG016	397	6	XP_001419361	40	9.00E-06	succinate:quinone oxidoreductase
RYG180	482	1	ABK22110	56	1.00E-13	ubiquinol-cytochrome c reductase complex
RYG131	170	1	DAA05918	65	1.00E-09	chlorophyll a-b binding protein of LHCI
RYG034	395	7	XP_001695344	91	3.00E-25	chlorophyll a-b binding protein of LHCII
RYG192	365	1	ACH55590	79	8.00E-39	cytochrome oxidase subunit 1
RYG236	808	1	ZP_03039658	31	1.00E-06	FAD linked oxidase domain protein
RYG088	654	3	XP_001694756	62	1.00E-46	the HCP family of iron-sulfur proteins
RYG125	245	1	XP_001695808	67	4.00E-16	mitochondrial ADP/ATP transporter
**2. Amino Acid Metabolism (4)**						
RYG197	179	1	XP_001702310	65	3.00E-10	arginine deiminase
RYG075	262	2	XP_001702649	74	3.00E-29	acetohydroxy acid isomeroreductase
RYG177	273	1	XP_002953664	47	2.00E-11	prolyl 4-hydroxylase alpha subunit-like protein
**3. Nucleotide Metabolism (2)**						
RYG057	296	2	YP_004701	67	9.00E-12	adenosine specific kinase
**4. Fatty Acid and Lipids Metabolism (5)**						
RYG143	382	1	XP_001693068	66	7.00E-09	omega-6 fatty acid desaturase
RYG265	187	1	XP_001448307	66	2.00E-12	long-chain acyl-CoA synthetase
RYG013	236	1	YP_001134657	61	1.00E-08	beta-ketoacyl synthase
RYG073	299	2	NP_001015794	53	8.00E-21	acylphosphate phosphohydrolase 2
**5. Carbohydrate Metabolism (12)**						
RYG042	178	2	XP_001692016	90	1.00E-13	ribose-5-phosphate isomerase
RYG176	389	1	BAB96757	83	2.00E-51	glucose-6-phosphate dehydrogenase 1
RYG224	202	1	AAZ30665	75	3.00E-16	fructose-bisphosphate aldolase
RYG187	180	1	XP_002511153	93	9.00E-19	alpha-1,4-glucan-protein synthase, UDP-forming
RYG082	296	3	XP_001693685	84	3.00E-22	UDP-glucose:protein transglucosylase
RYG148	308	2	NP_001142134	92	1.00E-34	reversibly glycosylated polypeptide
RYG158	318	1	YP_002886267	71	7.00E-38	isocitrate lyase
RYG246	536	1	NP_868980	36	9.00E-17	surface protein Sur1
**6. DNA/RNA Binding, Transcription and Translation (12,** **10 ribosomal genes were not listed)**						
RYG263	261	1	YP_002394584	100	2.00E-39	transposase
RYG195	278	1	ABK34467	59	7.00E-24	eukaryotic initiation factor 3e
**7. Protein Folding, Sorting and Degradation (16)**						
RYG019	377	2	XP_001701380	100	1.00E-35	ubiquitin family
RYG164	337	1	XP_002506123	60	9.00E-14	ubiquitin-conjugating enzyme E2
RYG112	338	12	XP_002155592	63	7.00E-27	FKBP-type peptidyl-prolyl cis-trans isomerase
RYG199	193	1	XP_002278975	69	5.00E-14	26S proteasome non-ATPase regulatory subunit
**8. Cell Cycle and Motility (3)**						
RYG141	261	1	XP_001450847	49	4.00E-10	cyclin
RYG181	306	1	ABR22558	90	3.00E-45	beta-tubulin
RYG276	276	1	XP_001700026	61	2.00E-22	alpha-SNAP
**9. Signal Transduction (1)**						
RYG184	674	1	XP_002529210	52	2.00E-42	signalosome subunit
**10. Chlorophyll and Polyketides Biosynthesis (25)**						
RYG109	565	23	XP_001701729	71	2.00E-30	coproporphyrinogen III oxidase
RYG234	253	1	XP_001697519	62	5.00E-20	glutamate-1-semialdehyde aminotransferase
RYG005	280	1	CAL52001	53	8.00E-10	polyketide synthase
**11. Function Unknown (666)**						

Numbers in parentheses represent the number of ESTs categorized into each group.

However, in addition to the putatively identified genes, ESTs for no-hit sequences with homologous sequences in the nr database (E-value >1e-5) and functionally unknown categories have a relatively higher abundance [29% in forward library (Table 2) and 87% in reverse library (Table 3)], which illustrates the current lack of genomic knowledge on green algae. These functionally non-characterized ESTs potentially represent previously unidentified genes involved in algal adaption to light stress and glucose starvation. Moreover, the same function could also be carried out by differentially regulated homologs. However, the uncharacterized genes need to be further analyzed to confirm their differential expression.

**Figure 3:**
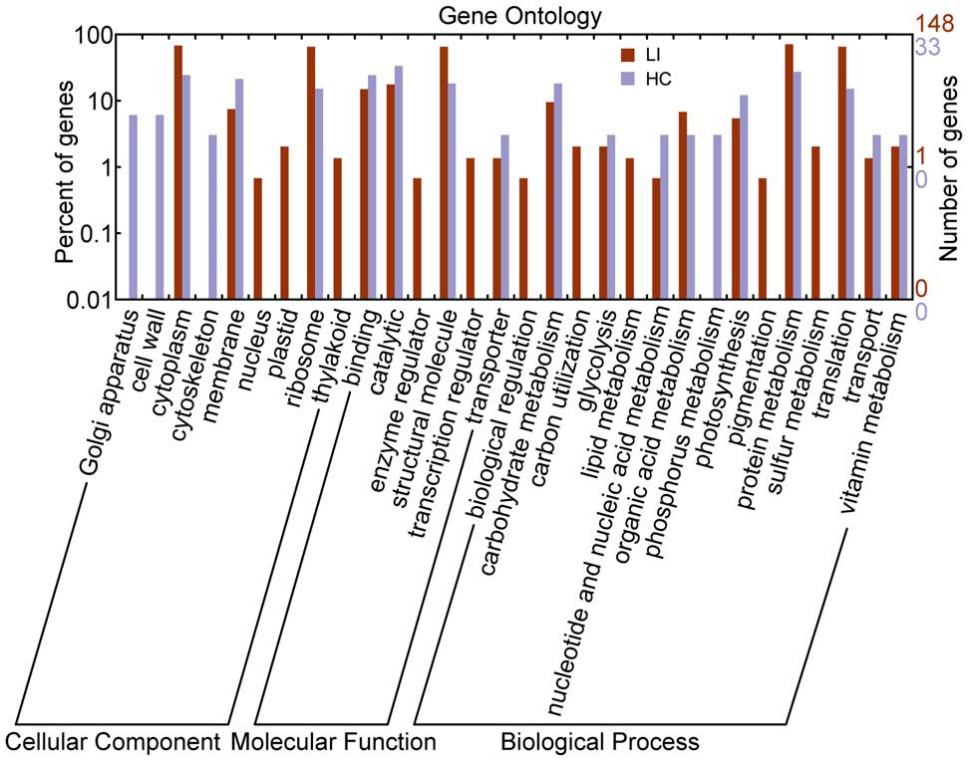
Gene Ontology (GO) annotation of genes obtained from the SSH libraries. GO predictions identified several categories based on the three terms cellular component, molecular function, and biological process, and were plotted by WEGO. LI represents forward library under the light-induced treatment; HC represents the reverse library under the heterotrophic culture process.

Functional analysis of the unigenes from the two libraries using Gene Ontology (GO) predictions identified several categories according to cellular component, molecular function, and biological process and were plotted using WEGO [33]. GO annotation coarsely identified the major categories of the differentially expressed genes involved in particular biological processes to assess the trends in their transcriptional regulation following glucose starvation and light induction (Figure 3). The annotation results show that the cellular component of light-induced cells were mainly located in ribosomes, the cytoplasm, and the cell membrane, which accounted for 75% of the total proportion. Likewise, cellular components of heterotrophic cells were also mainly located in the cytoplasm, cell membrane, and ribosome. The unique locations for the forward libraries were the plastids, thylakoids, the nuclei, and the cell wall, whereas the reverse libraries were in the cytoskeleton and the Golgi apparatus. The most commonly represented categories in the light-induced cells and the heterotrophic growth cells were those of structural molecules, followed by catalytic activity, and binding activities. The other molecular functions were represented at a lower extent. In terms of biological processes, the main ESTs of heterotrophic cells were related to carbohydrate metabolism, protein metabolism, and translation, particularly in phosphorus metabolism and nucleotide metabolism. Meanwhile, the most represented in the light-induced cells were translation, protein metabolism, organic acid metabolism, photosynthesis, carbohydrate metabolism, especially in carbon utilization, lipid metabolism, sulfur metabolism, and pigmentation. According to the information given by the GO annotation, the heterotrophic cells tended towards growth, development, and primary metabolic processes, whereas the light-induced *Chlorella* cells tended to be more active in response to environmental factors and self-protections.

### Confirmation of Differential Expression and Transcriptional Levels

About 50 upregulated and downregulated unigenes were preferentially chosen based on the putative annotations linking them to biological processes considered significant. Since the data suggested a large number of identified genes are as of yet uncharacterized, we also added 15 randomly selected functional unknown sequences for chosen. This step was performed to validate the results from SSH experiments. Primers for RT-PCR of these genes were designed by using Primer Premier 5 software. However, since the software produced the primer pairs of only 38 genes (including 9 function unknown genes) as shown in Table S1 and S2, RT-PCR analyses were performed for these genes (20 for semi-quantitative and 28 for real-time quantitative, 10 genes were overlapped). All the selected genes showed significant differential expression at all the six different time points. As shown in Figure S6, the mRNA abundance of the 10 genes from the forward library was notably induced in the light-treated samples, confirming that these genes were upregulated by light stress and glucose starvation. On the contrary, most the 10 genes from the reverse library were significantly downregulated when the *Chlorella* cells were exposed to light illumination relative to the cells by heterotrophic growth. These results indicated that the genes identified in our study are transcribed differentially under heterotrophy to photoautotrophy transition.

**Figure 4:**
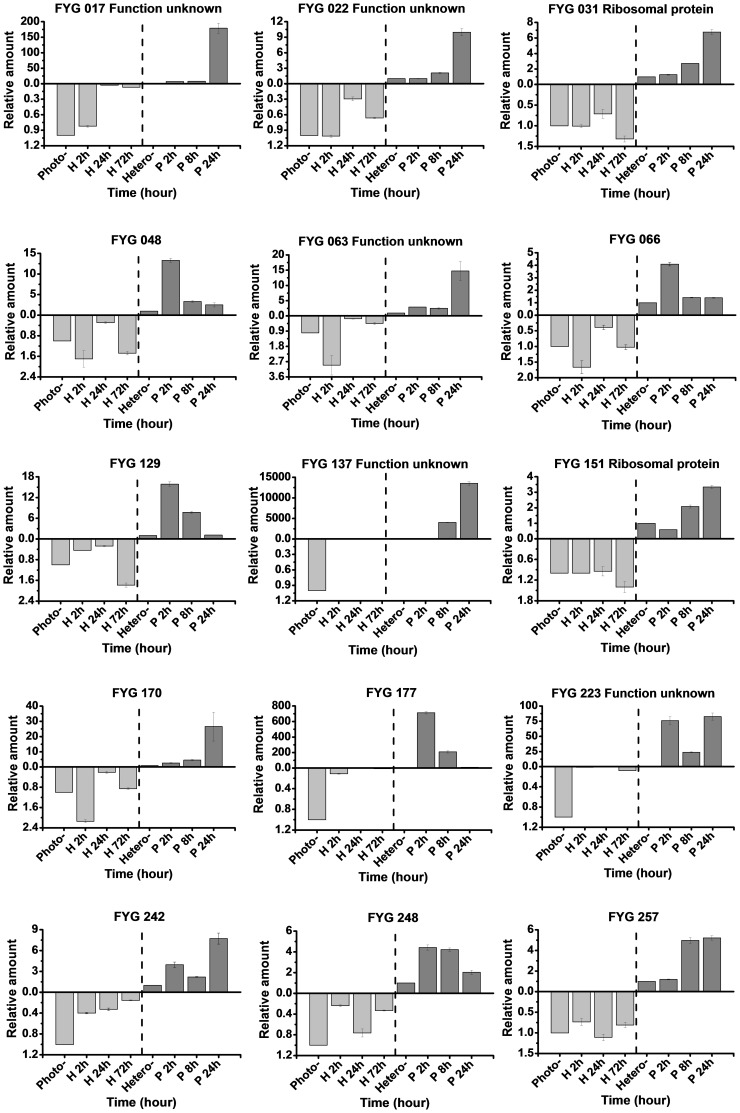
Time-course expression patterns of the selected differentially expressed genes from the forward SSH library. All expression values were normalized to the value of actin gene. Relative amount was calibrated based on the transcript amount of the corresponding gene in control (aliquots of the cDNA samples were mixed from different time points). Left of the dotted line represent the mixed photoautotrophic cells as control and heterotrophic cells as treatment. Right of the dotted line is just the opposite. Values are mean ± standard deviation obtained from three independent biological experiments. Hetero- and H: heterotrophy; Photo- and P: photoautotrophy.

**Figure 5:**
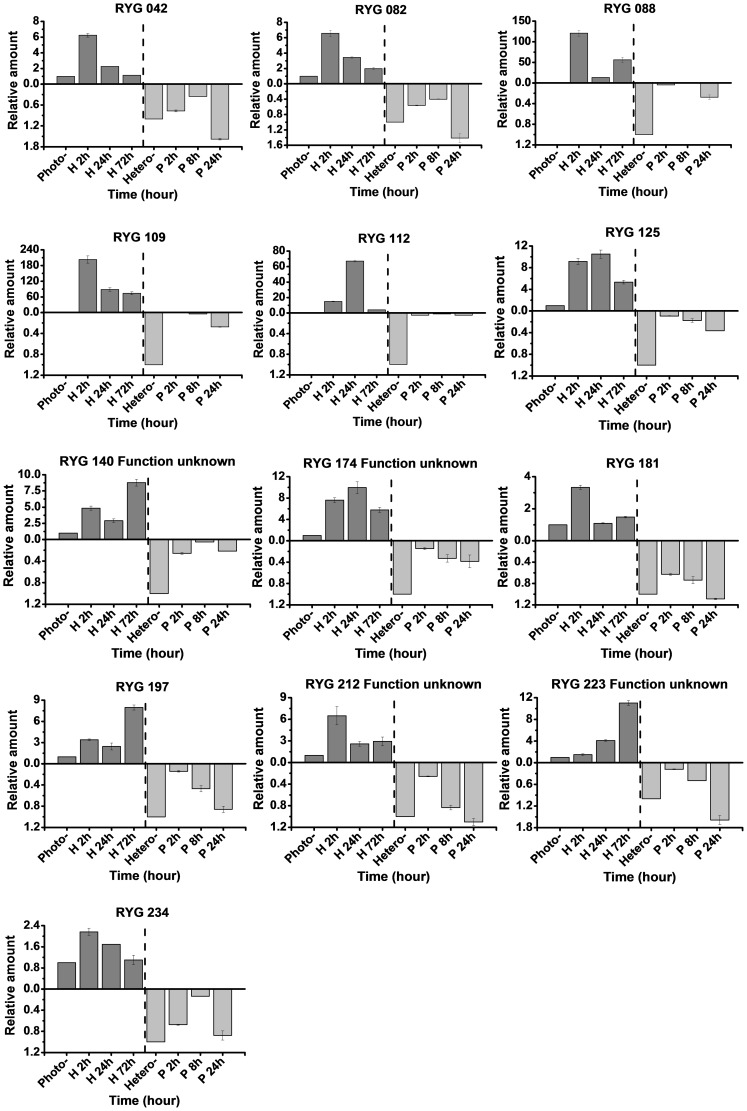
Time-course expression patterns of the selected differentially expressed genes from the reverse SSH library. All expression values were normalized to the value of actin gene. Relative amount was calibrated based on the transcript amount of the corresponding gene in control (aliquots of the cDNA samples were mixed from different time points). Left of the dotted line represent the mixed photoautotrophic cells as control and heterotrophic cells as treatment. Right of the dotted line is just the opposite. Values are mean ± standard deviation obtained from three independent biological experiments. Hetero- and H: heterotrophy; Photo- and P: photoautotrophy.

The time-course expression profiles of the 28 candidate genes were examined using materials sampled at different points of the culture stages to investigate how the aforementioned genes responded to glucose starvation and light stress (Figure 4 and 5). The real-time RT-PCR results revealed general considerations of transcript profiling of *C. pyrenoidosa* under diverse nutrition transition, with most of the light-induced genes exhibiting a fast response and reaching their maximum transcription levels at the first 24 h after light exposure. Most of the genes continued to increase until the end of the experiment. In the reverse library, differences among the expression profiles were also detected. Three genes showed continued upregulation in the late growing season in dark (RYG140, RYG197, RYG223, 72 h of heterotrophy), confirming the effect of glucose starvation on gene expression. In total, five genes remarkable increases in transcripts over 100 times during the culture process were observed (FYG017, FYG137, FYG177, RYG 088 and RYG109). Nevertheless, for all of the genes, the RT-PCR results were in reasonable agreement with the SSH experiments, which suggests that these genes were truly differentially expressed.

## Discussion

Microalgae have been extensively investigated and exploited because of their competitive nutritive bioproducts and biofuel production ability. However, the large-scale applications have still been limited by the low yield and high cost of microalgal biomass production. Photoautotrophic culture model is widely used in conventional commercial production process, yet its large area coverage, low productivity and high production cost are the main limiting factors. Heterotrophy can achieve considerable cell density and productivity. However, under heterotrophic conditions, cells are lacking in photosynthetically derived compounds (lower protein and pigment contents), leading the loss of its main advantages and practical application value [5]. In this study, we adopted the novel SHDP culture model to culture *Chlorella*, the contents of protein and chlorophyll were increased markedly within the first 8-h photoinduction (Figure 1), making the quality of the cells (protein and chlorophyll contents) obtained by SHDP close to the maximum values reported for purely photoautotrophically grown cells [22].

Although several studies have been carried out to explore the mechanisms of stress tolerance in microalgae [34]–[36], information on the genetic expression involved in their diverse trophic niche is limited. *Chlorella* is a particular green algal genus that can grow well heterotrophically and photoautotrophically. Thus, genes were screened to determine the distinct nutritional status of microalgae for the first time. *Chlorella* cultured via the SHDP strategy can also efficiently accumulate high-value bioproducts within several hours; thus, the identification of genes involved in the culture process might provide clues for molecular interpretation and will facilitate further improvement of these organisms and the novel technique used. SSH is one of the most powerful methods for identifying differentially expressed transcripts. In the present study, forward and reverse libraries were constructed and screened, which yielded nearly 3,000 positive clones. About 1,500 recombinant clones were subsequently sequenced, and 544 unigenes were identified as differentially expressed genes responsive to trophic transition. Based on the homology search and functional annotation, 238 (44%) unigenes were found to encode proteins with either known or putative functions. In addition, the better part of ESTs with non-homologous proteins checked against the databases has been identified in the two libraries. These ESTs could potentially represent the sequences of the 5′ and 3′ untranslated regions or novel species-specific genes that were previously poorly characterized, and may play an important role in the adaption of microalgae to glucose starvation and light stress. The differentially expressed genes obtained with significant protein homology were categorized into 12 groups according to their putative function, which imply their distinct trophic status leads to a dramatic response in gene expression involved in complex biological pathways. These genes may impart a variety of physiologic and biochemical changes in *Chlorella* by modulating metabolic flux trends, providing a crucial molecular basis for the transition from heterotrophy to photoautotrophy leading to the reassortment of intracellular components.

In the category “energy metabolism,” 57 ESTs individually encoded 21 proteins. The genes that encoded the corresponding photosystem proteins (3 unigenes), light-harvesting complexes (2 unigenes), photosynthetic electron transport proteins (1 unigene), and chloroplast Calvin cycle enzymes (4 unigenes) were found in the forward library, such as oxygen-evolving enhancer protein, chlorophyll a-b binding protein, plastocyanin oxidoreductase iron–sulfur protein, and ribulose-1,5-bisphosphate carboxylase oxygenase large/small subunit. These proteins suggest the robust ability of *Chlorella* to restart photosynthetic function to protect heterotrophically grown cells from photodamage under sudden light stress. Likewise, the upregulation of these proteins in response to light stress has also been reported in higher plants and algae [37], [38]. By contrast, the heterotrophic cells were expectedly deprived of photosynthetic performance; thus, few genes related to photosynthetic pathways were found in the reverse library. However, two special cases were found, namely, the antenna proteins of light-harvesting complexes (RYG131 and RYG034), which showed enhanced expression in response to heterotrophy. Plant and green algal light-harvesting complexes are composed of more than 20 different antenna proteins associated with photosystems I and II [39]. Although the function of antenna proteins in darkness-grown cells was not explored, the relatively increased expression indicates their importance in heterotrophic *Chlorella*. In unicellular green algae *Dunaliella tertiolecta*, an increase in the relative abundance of chlorophyll a/b light-harvesting complex mRNA was also found following a shift from high light to darkness and from high light to low light [40]. LaRoche *et al* proposed that changes in energy balance brought about by a change in light intensity may control a regulatory factor acting to repress chlorophyll a/b binding protein mRNA expression [40]. Furthermore, a unigene that encodes glutamate dehydrogenase (GDH), was also found in the forward library. GDH is an important intermediate enzyme between carbon and nitrogen metabolism and it functions in multiple directions, depending on the environment and the stress [41], [42]. In transgenic plants, the overexpression of microbial GDH reportedly confers improved tolerance to herbicides, drought, and pathogenic infections [43]. Thus, the upregulation of the gene might be important for the acquisition of nutritional transition tolerance. In the reverse library, several ESTs that encode oxidoreductases (RYG016, RYG180, RYG192, and RYG236) involved in the respiratory electron-transport chain were enriched congruously. The enhanced expression of genes related to cellular respiration indicates exuberant anabolism in the darkness-grown *Chlorella*, which is in line with the competitive cellular growth rate during the heterotrophic process compared with that in light-induced cells.

The “DNA/RNA binding, transcription and translation” category was the most abundant among the genes identified in the forward library, especially those that encode ribosomal proteins, taking over nearly half of the sequenced clones (342 ESTs). In addition, four unigenes individually encoded proteins homologous to transcription factor BTF3, retrotransposon protein, RNA helicase, and elongation factor 1-alpha. Previous studies confirmed that some ribosomal protein genes are regulated by different stress environments, including high salinity and cold stress [36], [44], [45]. Organisms have evolved a translation machinery referred to as *de novo* protein synthesis to cope with stress. In *Chlorella*, the increase in the number of transcripts of various ribosomal protein subunits may be responsible for the reboot of the photosynthetic apparatus and related enzymes after they are returned under light. In higher plants, transcription factor BTF3 is required for RNA polymerase II-dependent transcription. The downregulation of BTF3 expression reduces the chloroplast size and the chlorophyll content [46]. Therefore, the upregulation of BTF3 activity may contribute to chloroplast development in *Chlorella* to accommodate light stress. Plant retrotransposons are reportedly minimized by maintaining a quiescent state during normal growth and development but could be stress-induced under life-threatening situations [47]. Although the function of retrotransposon proteins in trophic transition remains unclear, the transcriptional increase may reflect a survival strategy in *Chlorella* during the transition from heterotrophy to photoautotrophy. RNA helicases are ubiquitous enzymes that function as molecular motors that rearrange duplex RNA secondary structures [48]. The helicases might be involved in regulating plant growth and development under stress conditions by regulating some stress-induced pathways [49], [50]. In the present study, the mRNA induction of RNA helicase in the forward library may act as a potential regulator that buffers the sudden shock under glucose starvation and light stress. Two other ESTs that individually encode transposase (RYG263) and eukaryotic initiation factor 3e (RYG195) were found in the reverse library, despite the relationships between the two genes and heterotrophic growth have not yet been reported.

In the present study, the diverse expression of 10 genes that encode putative protein with functions related to amino acid metabolism and protein folding, sorting, and degradation were identified in both libraries. Among them, diaminopimelate epimerase plays a critical role in the biosynthetic pathway that converts aspartate into lysine. Further research into the enhancement of the metabolic flux through this pathway could increase the essential amino acid yield in important crops, thereby improving their nutritional value [51]. Another notable unigene is arginine deiminase, a key enzyme that participates in arginine and proline metabolism. In bacteria, arginine deiminase expression is regulated by various environmental factors and growth phases [52]. The upregulation of this enzyme was reported in response to growth under both temperature and salt stress conditions [53]. As demonstrated in the current work, the increased transcription level of arginine deiminase produces ammonia and ATP, thereby allowing the darkness-grown cells to survive and gain an energy advantage. The induced expression of ESTs that encode acetohydroxy acid isomeroreductase (AAIR), the prolyl 4-hydroxylase alpha subunit-like protein (P4HA), ubiquitin-conjugating enzyme, FKBP-type peptidyl-prolyl cis–trans isomerase, and the 26S proteasome non-ATPase regulatory subunit were also characterized in the reverse library. AAIR is an important enzyme in the biosynthetic pathway of branched-chain amino acids in plants and microorganisms, whereas P4HA encodes a component of prolyl 4-hydroxylase, a key enzyme that catalyzes the formation of 4-hydroxyproline essential to the proper three-dimensional folding of newly synthesized procollagen chains. These functions, which are potentially necessary for heterotrophic *Chlorella*, may help cells grow well in the dark [54], [55].

A previous study demonstrated that the fatty acids profile of *Chlorella* changes significantly after light induction [5]. In the present study, we found two distinct omega-6 fatty acid desaturases (FAD6) individually expressed in the two libraries. FAD6 is involved in the desaturation of fatty acids. Two FAD6 isoenzymes were identified in *Arabidopsis* [56], [57]. The upregulation of chloroplastic FAD6 isotype in response to freezing stress has been reported in *C. vulgaris* [36]. Two other ESTs that are related to fatty acid synthesis and activation were identified from the reverse library, namely, beta-ketoacyl synthase (KAS) and long-chain acyl-CoA synthetase (LC-FACS). KAS catalyzes the condensation of malonyl-ACP with the growing fatty acid chain, whereas LC-FACS plays a role in the pre-step reaction for the β-oxidation of fatty acids and it can be incorporated in phospholipids. Although the functions of these enzymes in the diverse trophic traits of *Chlorella* remain unclear, induction or repression of these genes may be important in maintaining cell membrane performance.

Our study revealed the upregulation of genes involved in the pentose phosphate pathway (RYG042 and RYG176), gluconeogenesis (RYG224), and the glyoxylate cycle (RYG158) in the reverse library, which suggests the global vigorous expression of central carbon metabolism in the heterotrophically grown cells. In plants, the pentose phosphate pathway is usually stimulated by stress conditions, which supplies reductive intermediates to cells by maintaining the NADPH levels [58]. The transcription of the proteins in the pentose phosphate pathway was also upregulated in S-deprived *Chlamydomonas* [17]. The glyoxylate cycle serves as the bypass pathway of the TCA cycle and is able to synthesize acetyl-CoA products from the degradation of fatty acids [59], which is especially important for cells that use oils as nutrient sources. In the present study, the induced expression of these metabolism-related genes may be linked to the high demand for metabolic intermediates for heterotrophic growth and biomass production. The plant cell wall is located outside the cell membrane and provides cells with structural support and protection. The cell wall is significantly important in cell enlargement, which plays a critical role in growth and development [60]. The upregulated expression of ESTs associated with cell wall metabolism, such as UDP-forming alpha-1,4-glucan-protein synthase, UDP-glucose:protein transglucosylase (UPTG), and reversibly glycosylated polypeptide (RGP) related to heterotrophic cells were identified in this study. These enzymes have a similar function in the synthesis of cell wall polysaccharides in plants [61], [62]. A correlation was found between the UPTG transcript levels and the growing status of potato tissues wherein the cell wall components were actively synthesized [61]. A possible explanation for the differential expression of cell wall–related genes is that *Chlorella* growth and development under heterotrophy and under light induction alter the structure and composition of the cell walls. Thus, the transcripts of genes related to cell wall polysaccharide synthesis are also possibly changed.

Aside from the aforementioned genes identified in both the forward and reverse libraries, we also found several representations of ESTs involved in cell cycle regulation and motility, signaling, chlorophyll biosynthesis, transport, and stress defense (Tables 2 and 3). Among them, the transcription levels of unigenes that encode thiazole biosynthetic enzyme and coproporphyrinogen III oxidase remarkably increase, both with more than 20 ESTs assembled. The thiazole biosynthetic enzyme and its plant homolog appear to be dual functional proteins with roles in thiamine biosynthesis and mitochondrial DNA damage tolerance [63], [64]. Therefore, its function may help *Chlorella* cells become more robust during trophic transition. Plants and algae under high light stress have to adapt to oxidative stress, which is associated with the generation of reactive oxygen species [37]. Therefore, increasing ROS scavenging capacity by enhancing the expression of ROS scavengers is essential to cells (Figure 2B and 2C). Corresponding to this, an EST that encodes the putative glutathione peroxidase (GPX) was found in the forward library. GPX represents an enzyme family with peroxidase activity that mainly functions in protecting the organism from oxidative damage, thereby stabilizing the light-induced *Chlorella* cells and maintaining intracellular activity under sudden light treatment.

In summary, the results described herein provide the valuable first insights into the molecular foundation associated with microalgal trophic transition, as well as glucose starvation and light stress. This study also provides an important basis for selecting candidate genes related to the SHDP process in *Chlorella*. These identified genes encompass a range of physiologic processes with pathways specific to both heterotrophic and phototrophic metabolism. Further studies are currently in progress, including screening for differentially expressed genes and pathways and clarifying their functional significance at the whole genome and transcriptome levels. These studies will facilitate further investigations that aim to improve the lipid and nutrient production in *Chlorella*.

## Supporting Information

Figure S1 **Analysis of the subtraction efficiency using PCR.** The subtracted and unsubtracted pools of cDNA from forward and reverse libraries were amplified by using primers for the constitutively expressed beta-actin gene. A: forward library; B: reverse library; M: marker.
(DOCX)

Figure S2 **Detection of inserted fragments using colony PCR.** The insets of 20 randomly selected clones in the two subtracted libraries were tested.
(DOCX)

Figure S3 **Length and distribution of sequenced reads among two SSH libraries.** The vector sequences were included in the results. A: forward library; B: reverse library.
(DOCX)

Figure S4 **Length and distribution of ESTs and assembled unigenes among two SSH libraries.** A: EST lengths without vector sequences from the forward library; B: unigene lengths identified from the forward library; C: EST lengths without vector sequences from the reverse library; D: unigene lengths identified from the reverse library.
(DOCX)

Figure S5 **COGs-based functional classification of the differentially expressed genes.** A: genes from the forward library; B: genes from the reverse library.
(DOCX)

Figure S6 **Time-course expression patterns of the selected differentially expressed genes from the two SSH libraries.** Semiquantitative RT-PCR was carried out using *actin* as the internal control. HC: heterotrophic culture; LP: Light-induced process.
(DOCX)

Table S1 **Sequences of primer pairs used for semi-quantitative RT-PCR.**
(DOCX)

Table S2 **Sequences of primer pairs used for real-time quantitative RT-PCR.**
(DOC)

Table S3 **Functional annotation and bioinformatics analysis of the properly assembled contigs and singletons from the forward library.** The ESTs with significant database threshold E-value of at least 1e-5 were classified into putative functional categories.
(XLS)

Table S4 **Functional annotation and bioinformatics analysis of the properly assembled contigs and singletons from the reverse library.** The ESTs with a significant database threshold E-value of at least 1e-5 were classified into putative functional categories.
(XLS)
