# Supplementary figures and images for: Suppression Subtractive Hybridization Reveals Transcript Profiling of Chlorella under Heterotrophy to Photoautotrophy Transition
Source: PLoS One. 2012 Nov 29;7(11):e50414. doi: 10.1371/journal.pone.0050414 (PMC3510161; doi:10.1371/journal.pone.0050414)

Figure S1


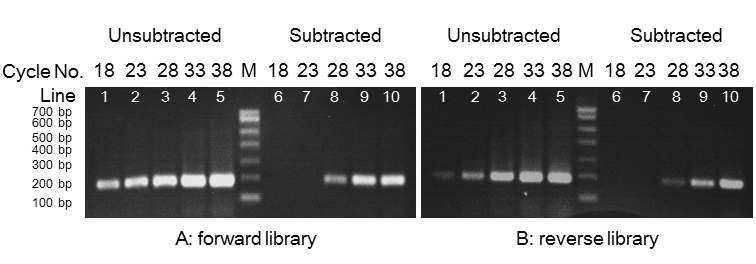

Supplement: Figure S1 — Analysis of the subtraction efficiency using PCR. The subtracted and unsubtracted pools of cDNA from forward and reverse libraries were amplified by using primers for the constitutively expressed beta-actin gene. A: forward library; B: reverse library; M: marker. (DOCX) [file pone.0050414.s001.docx]

**Figure S2**


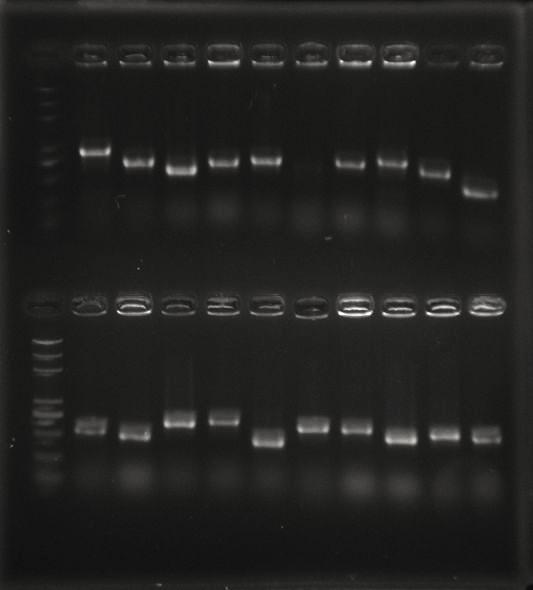


**500**

**750**

**1000**

**2000**

**3000**

**5000**

**Lane/bp**

**Lane/bp**

**500**

**750**

**1000**

**3000**

**5000**

**2000**

Supplement: Figure S2 — Detection of inserted fragments using colony PCR. The insets of 20 randomly selected clones in the two subtracted libraries were tested. (DOCX) [file pone.0050414.s002.docx]

**Figure S3**


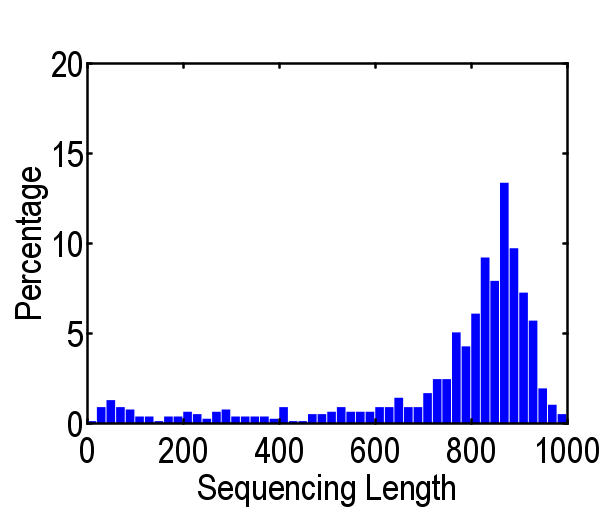


**A**


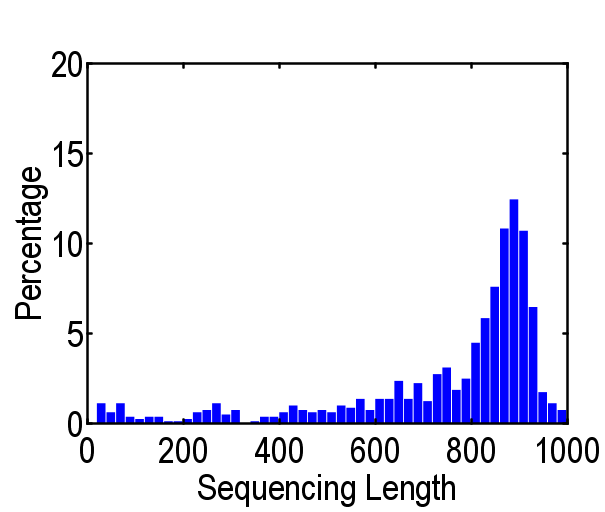


**B**

Supplement: Figure S3 — Length and distribution of sequenced reads among two SSH libraries. The vector sequences were included in the results. A: forward library; B: reverse library. (DOCX) [file pone.0050414.s003.docx]

**Figure S4**


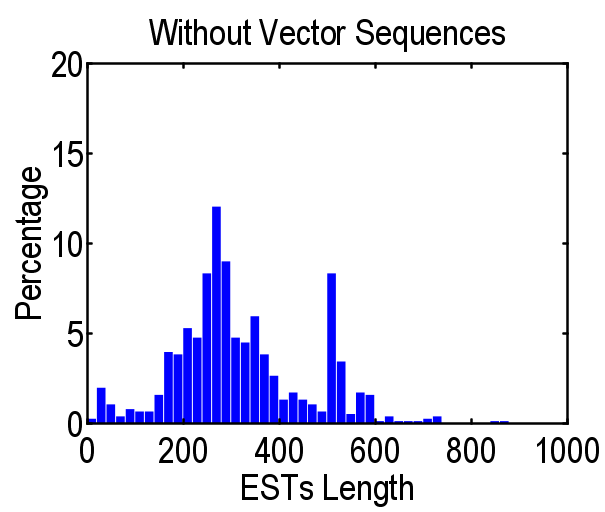

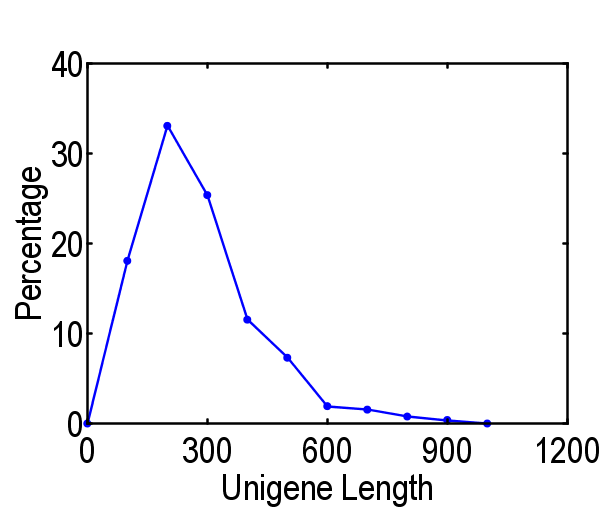


**D**

**C**

**B**

**A**


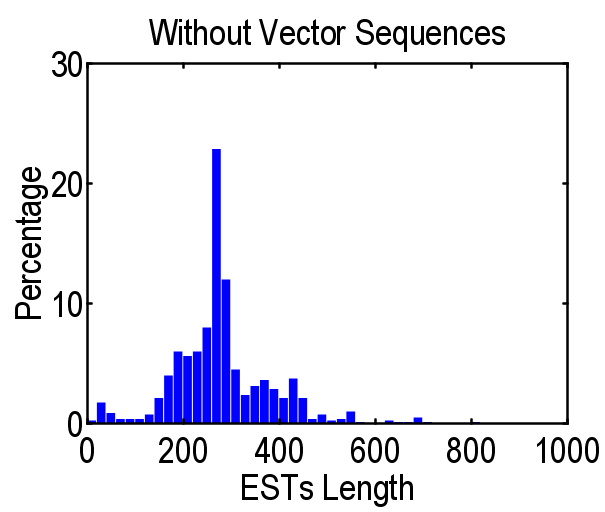

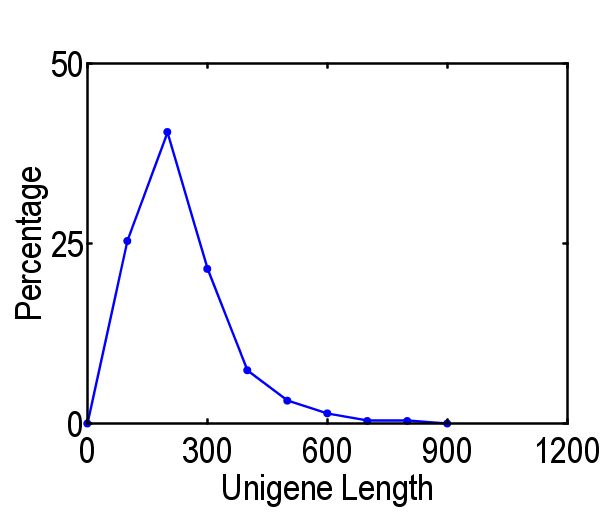

Supplement: Figure S4 — Length and distribution of ESTs and assembled unigenes among two SSH libraries. A: EST lengths without vector sequences from the forward library; B: unigene lengths identified from the forward library; C: EST lengths without vector sequences from the reverse library; D: unigene lengths identified from the reverse library. (DOCX) [file pone.0050414.s004.docx]

**Figure S5**


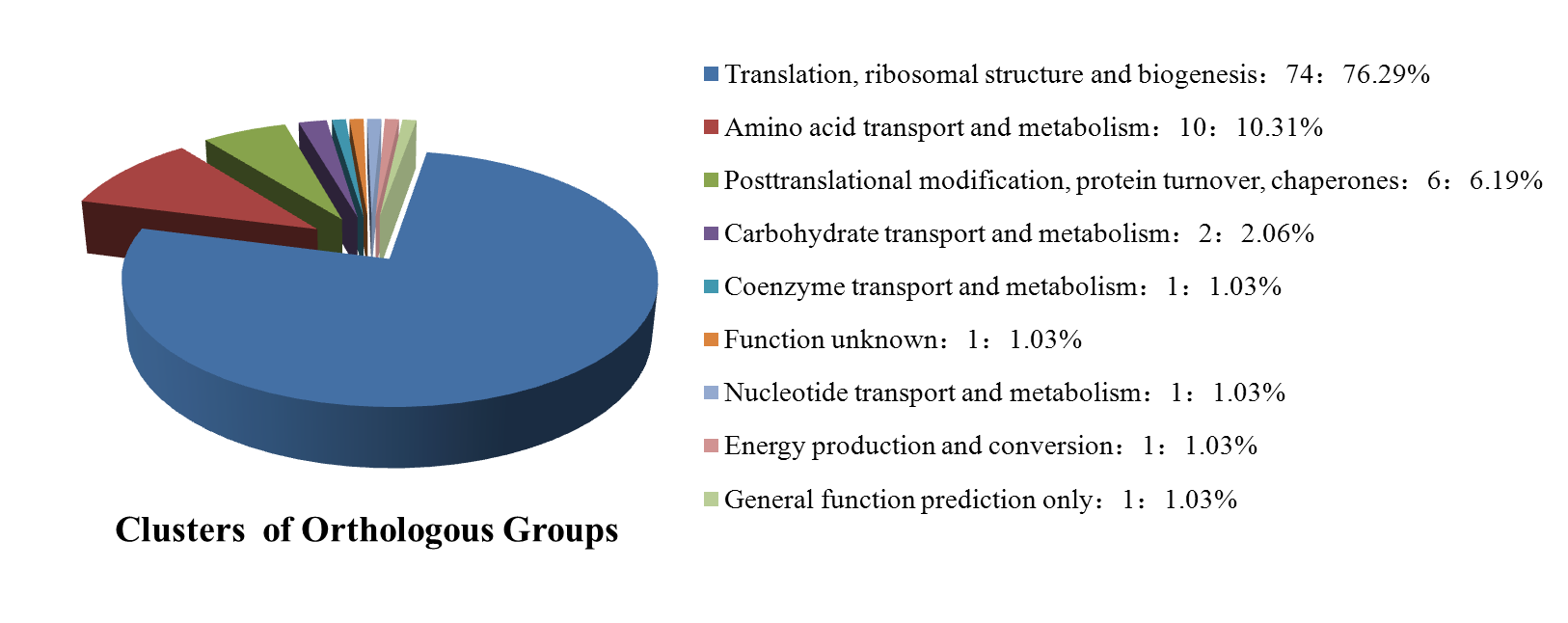


**A**


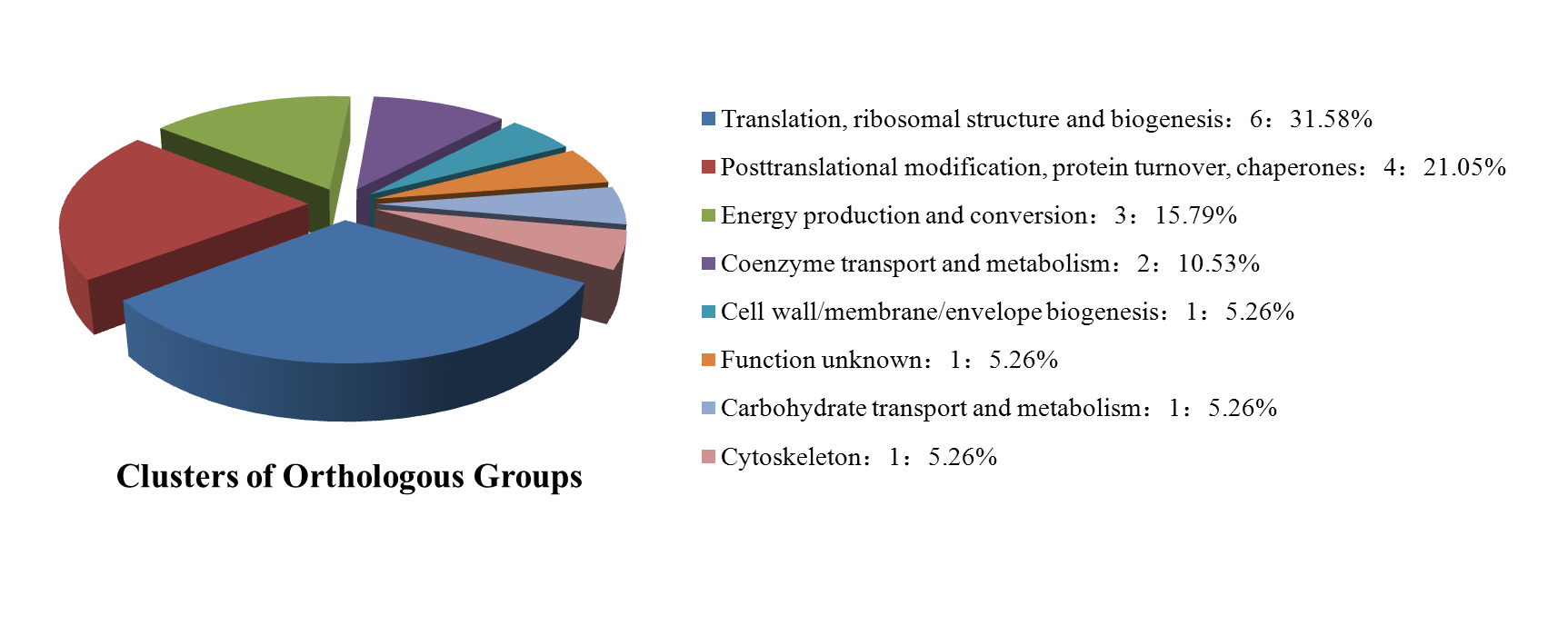


**B**

Supplement: Figure S5 — COGs-based functional classification of the differentially expressed genes. A: genes from the forward library; B: genes from the reverse library. (DOCX) [file pone.0050414.s005.docx]
