# Supplementary material for: Suppression Subtractive Hybridization Reveals Transcript Profiling of Chlorella under Heterotrophy to Photoautotrophy Transition
Source: PLoS One. 2012 Nov 29;7(11):e50414. doi: 10.1371/journal.pone.0050414 (PMC3510161; doi:10.1371/journal.pone.0050414)
Supplement: Figure S6 — Time-course expression patterns of the selected differentially expressed genes from the two SSH libraries. Semiquantitative RT-PCR was carried out using actin as the internal control. HC: heterotrophic culture; LP: Light-induced process. (DOCX) [file pone.0050414.s006.docx]

HC

LP

LP

HC

24h

8h

2h

24h

8h

2h

24h

8h

2h

24h

8h

2h


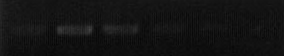

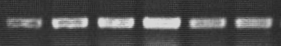


RYG034

FYG048


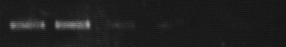

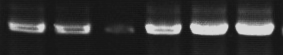


FYG062

RYG082


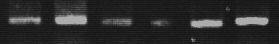

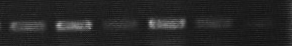


FYG066

RYG109


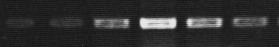

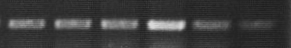


FYG127

RYG112


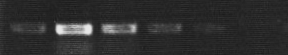

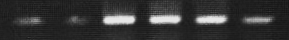


FYG129

RYG125


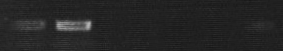

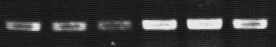


FYG152

RYG141


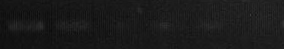

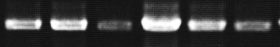


FYG177

RYG176


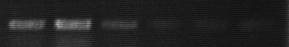

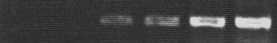


FYG201

RYG181


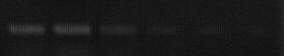

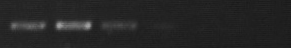


FYG215

RYG224


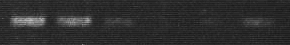

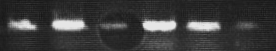


FYG248

RYG276


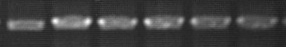


Actin
