# Supplementary material for: Suppression Subtractive Hybridization Reveals Transcript Profiling of Chlorella under Heterotrophy to Photoautotrophy Transition
Source: PLoS One. 2012 Nov 29;7(11):e50414. doi: 10.1371/journal.pone.0050414 (PMC3510161; doi:10.1371/journal.pone.0050414)
Supplement: Table S1 — Sequences of primer pairs used for semi-quantitative RT-PCR. (DOCX) [file pone.0050414.s007.docx]

**Table S1. Sequences of primer pairs used for semi-quantitative RT-PCR**

| **Gene ID** | **Annotation** | **Forward primer (5’-3’)** | **Reverse primer (5’-3’)** | **Annealing temperature (℃)** | **Product size (bp)** | |
| --- | --- | --- | --- | --- | --- | --- |
| RYG125 | mitochondrial ADP/ATP transporter | AAGCCCTTTGTGAACGG | TTCTCGCCCAGTTGGAT | 56.0 | 95 |  |
| RYG034 | chlorophyll a-b binding protein of LHCII | ACCAAGTTTGTGCCCAG | GCAAGGGAGGGTCATAGAT | 55.5 | 132 |  |
| RYG224 | fructose-bisphosphate aldolase | ATCAATGCCCTGGGTCTC | GACAAGGACATCGTGCCT | 57.0 | 90 |  |
| RYG082 | UDP-glucose:protein transglucosylase | AGGTGGTAGGGCTGGAAGA | AGGTCATCGGCAAGCACT | 59.0 | 199 |  |
| RYG112 | FKBP-type peptidyl-prolyl cis-trans isomerase | TCGCCAACCTTCATCTGC | CCCTTCCCTCGCAATCAT | 60.5 | 225 |  |
| RYG176 | glucose-6-phosphate dehydrogenase | AGGTGGTTCTGGATGACG | GTTTGTGATGCGGTTTGC | 56.5 | 159 |  |
| RYG141 | cyclin | GCCTCCAAGCACGAAGA | GGGTGTTGATGCGGAAG | 58.0 | 136 |  |
| RYG181 | beta-tubulin | AGGTGGACGAGCAGATG | CACAAAGGTGGCAGACA | 55.0 | 131 |  |
| RYG109 | coproporphyrinogen III oxidase | TGCCTCACACCCAGTTGT | CCGCTACACCGAGTTCAA | 58.5 | 200 |  |
| RYG276 | alpha-SNAP | GCAGGTTGTTGTCCACG | TGTTCTACGAGCAGGCG | 56.5 | 165 |  |
| FYG201 | glutathione peroxidase | GCCTACAGACAGAGGTGCTC | ACTGCCGAAATCAAGTGG | 57.0 | 197 |  |
| FYG048 | chloride ion channels family protein | TGAACTTGGTCACCTCGC | GGTTTGGGCAGACACAGA | 58.0 | 125 |  |
| FYG127 | ribosomal protein L44 | CTGGATGTGGAAAGAGCG | CGACAAGAAGCAGAAGGG | 57.0 | 133 |  |
| FYG215 | nucleoside diphosphate kinase group I | TCGGCGTAGTGAGACTCTG | GCTGACCTTTGTGATGCTC | 56.0 | 147 |  |
| FYG129 | diaminopimelate epimerase | ACCTCAACAAACTCCGTGT | CACCTGGCTGATGACCT | 55.0 | 168 |  |
| FYG248 | glyceraldehyde-3-phosphate dehydrogenase | ATCCTGGGCGTGAGCGA | CGGCGTCAATGGTGGAG | 62.0 | 76 |  |
| FYG062 | light harvesting complex a | CCGTTGTTGATGAAGTGGT | GCCTGAAGCAGAAGGAGAT | 56.5 | 152 |  |
| FYG152 | chlorophyll a-b binding protein of LHCII | ACGGCTCTGCCAACTTCG | CATCAGCACCACCTGGGTC | 61.5 | 146 |  |
| FYG177 | glutamate dehydrogenase (NADP+) | TCGCCGAGAACTTGTGC | GCTGAACGAGGAGGATGC | 59.0 | 107 |  |
| FYG066 | fructose-1,6-bisphosphate aldolase | CAGGTTCAGGGTGGACTC | TGAGCACGCCAAGAAGT | 56.0 | 139 |  |
| Internal control | Actin | CATCTACGAGGGCTACGCGCT | GAGGTCCTTGCGGATGTCCAC | 63.5 | 388 |  |
