# Supplementary material for: Suppression Subtractive Hybridization Reveals Transcript Profiling of Chlorella under Heterotrophy to Photoautotrophy Transition
Source: PLoS One. 2012 Nov 29;7(11):e50414. doi: 10.1371/journal.pone.0050414 (PMC3510161; doi:10.1371/journal.pone.0050414)
Supplement: Table S2 — Sequences of primer pairs used for real-time quantitative RT-PCR. (DOC) [file pone.0050414.s008.doc]

**Table S1. Sequences of primer pairs used for real-time quantitative RT-PCR**

| **Gene ID** | **Annotation** | **Forward primer (5’-3’)** | **Reverse primer (5’-3’)** | **Annealing temperature (℃)** | **Product size (bp)** |
| --- | --- | --- | --- | --- | --- |
| FYG017 | Function Unknown | CATCGCAGGCTTCCTCAAGGTG | GTCATGATAGCAGTGGCAGGACG | 59.0 | 225 |
| FYG022 | Function Unknown | GTTTATCGTGATCTCCGCTGTG | CAGCAGCAGCAGCCTGCTTGAC | 57.5 | 153 |
| FYG031 | Ribosomal protein | CAGCACCGGCTCAAACACCTTC | GGACGTTGCGGATTCACCAGC | 57.5 | 178 |
| FYG048 | chloride ion channels protein | CGGGAGCAGCGGACCTTGAACTT | GGCAGACACAGACAAGATGCCCAA | 59.0 | 135 |
| FYG063 | Function Unknown | GAGGATGGCCTTGTGTCGTTTGG | CAAGGCAGGGTGCAGGGACAAG | 60.0 | 242 |
| FYG066 | fructose-1,6-bisphosphate aldolase | GCAGCATCTTCAGGGTGTAGGA | CATCCTGCTGAAGCCCTCCAT | 58.5 | 95 |
| FYG129 | diaminopimelate epimerase | CAGCACCTCAACAAACTCCGTG | CATCAAGGTGGACGAGCTGGA | 58.0 | 100 |
| FYG137 | Function Unknown | ATTGAGGGCGTCTGGTATGCGC | ACACTCGTATGATACAGGCATGGA | 60.0 | 187 |
| FYG151 | Ribosomal protein | GACCTGCGGGACCTCTGGATT | TCACCACGTCCTTGCCGCTGAA | 58.5 | 166 |
| FYG170 | proliferation associated protein | CCGAGGAGACTCTGGCAAATCC | ACGCAGTTGTTGACGGAGATGCA | 59.5 | 232 |
| FYG177 | glutamate dehydrogenase (NADP+) | GCTCGCCGAGAACTTGTGCACAG | CCAGAACGAGCTGAACGAGGAG | 61.5 | 118 |
| FYG223 | Function Unknown | TACATGGCACAAGTGCGTCCAGC | CCCATAACCTTCAACCAGCAGTC | 58.0 | 264 |
| FYG242 | oxygen-evolving enhancer protein of photosystem II | GGTGAGGGCTTCGCTGTGCTG | TCGATGTTGCCCTTGCCGACCTT | 58.0 | 149 |
| FYG248 | glyceraldehyde-3-phosphate dehydrogenase subunit | TGAGCGACGAGCCCCTGGTGT | GCCACCACCTTGACCATGTCGT | 57.5 | 109 |
| FYG257 | psaG, subunit V of photosystem I | GGTGGACTTTGCCTACAACCTGC | CCTTGCCCTTGGTGATGGCGAT | 55.0 | 101 |
| RYG042 | ribose-5-phosphate isomerase | TGCAGCGGCTGCAGAACCTGC | CCACAATGTAGTTGGAGTTGTCG | 61.5 | 102 |
| RYG082 | UDP-glucose:protein transglucosylase | ACGATGATGAGGTGGTAGGGC | GGTCTCTAGCAGCTATGCCGAC | 58.0 | 127 |
| RYG088 | the HCP family of iron-sulfur proteins | GTGTTTGGCACCGACGTCAACG | CAGGCTGTCTAGGCATTGGCCA | 59.0 | 251 |
| RYG109 | coproporphyrinogen III oxidase | CAAGGGTAACGGTGAGAGCGGT | CAGCTGGCGCTACGACTACCAG | 60.0 | 189 |
| RYG112 | FKBP-type peptidyl-prolyl cis-trans isomerase | CTCGCCAACCTTCATCTGCATC | GGAAGTTTGACTCTTCTCGCGA | 58.5 | 110 |
| RYG125 | mitochondrial ADP/ATP transporter | GCCAAGCCCTTTGTGAACGGC | CAGTTGGATGCGCACCTTGACC | 58.5 | 90 |
| RYG140 | Function Unknown | GGCACGTTCACTCTCACCTCTCA | CCTCCTGCTCAATCTCCAGCTC | 58.0 | 229 |
| RYG174 | Function Unknown | GTACTTGGGCAGCTTCATGGACT | CTCTGCGCAGCACATATGTCGAC | 58.0 | 155 |
| RYG181 | beta-tubulin | CCGCATGTCCTCCAAGGAGGT | CGGTGGTGTTGCCCACAAAGG | 60.5 | 161 |
| RYG197 | arginine deiminase | GGGAGTAGTCGATGCACTGCAC | GTACGGCTGCAACTGCCTCAACC | 60.0 | 122 |
| RYG212 | Function Unknown | CAGTGGAGCAGTTCTATGAGGAG | AGAGTCATGGGAACGGAGGAGC | 60.0 | 193 |
| RYG223 | Function Unknown | GCAGAGCGCAGCCAGAGAGAG | TGCGACGGCACCATCACCCACAT | 58.0 | 236 |
| RYG234 | glutamate-1-semialdehyde aminotransferase | GCACCTGGACAAGATCACCAAGC | CAGAAGAAGAAGCCAAACATGCCG | 58.5 | 114 |
| Internal control | Actin | GCTCAACTCCTCCACGCT | GTCCTTGCGGATGTCCAC | 60.5 | 187 |
